# Supplementary material for: Deep Learning Predicts Hematopoietic Stem Cell Aging From 3D Chromatin Images
Source: Aging Cell. 2026 Sep 27;25(10):e70656. doi: 10.1111/acel.70656 (PMC13617143; doi:10.1111/acel.70656)
Supplement: Supplementary file 1 [file ACEL-25-e70656-s001.pdf]

Table 1: Performance Metrics of ChromAgeNet over the 5 cross-validation folds at 2D XY slide level.

Fold	Accuracy	Precision	Recall	AUC	F1
1	0.69	0.63	0.68	0.76	0.66
2	0.62	0.54	0.81	0.70	0.65
3	0.68	0.65	0.62	0.73	0.63
4	0.65	0.60	0.69	0.72	0.64
5	0.65	0.59	0.70	0.71	0.64
All mean	0.66	0.60	0.70	0.72	0.64
All std	0.03	0.04	0.07	0.02	0.01
All CI	(0.63, 0.68)	(0.57, 0.64)	(0.64, 0.76)	(0.70, 0.74)	(0.64, 0.65)

Table 2: Performance Metrics of ChromAgeNet over the 5 cross-validation folds at nucleus level using soft-voting.

Fold	Accuracy	Precision	Recall	AUC	F1
1	0.70	0.64	0.74	0.81	0.69
2	0.60	0.53	0.90	0.74	0.66
3	0.72	0.70	0.64	0.78	0.67
4	0.71	0.65	0.80	0.76	0.72
5	0.68	0.61	0.80	0.76	0.69
All mean	0.68	0.63	0.78	0.77	0.69
All std	0.05	0.06	0.09	0.03	0.02
All CI	(0.64, 0.72)	(0.57, 0.68)	(0.70, 0.86)	(0.75, 0.79)	(0.67, 0.70)

Table 3: Performance Metrics of Feature Selection Methods for the classification of manually-extracted features. Shown as Average ± Std (CI).

Method	Accuracy	Precision	Recall	AUC	F1
EMBED LR	0.68 ± 0.03 (0.65, 0.71)	0.69 ± 0.04 (0.65, 0.72)	0.69 ± 0.06 (0.64, 0.73)	0.68 ± 0.03 (0.65, 0.71)	0.68 ± 0.03 (0.65, 0.71)
EMBED RF	0.70 ± 0.05 (0.66, 0.75)	**0.70 ± 0.06 (0.65, 0.75)**	0.70 ± 0.05 (0.66, 0.75)	0.70 ± 0.05 (0.66, 0.75)	0.70 ± 0.05 (0.66, 0.75)
EMBED XGB	0.69 ± 0.05 (0.65, 0.73)	0.68 ± 0.05 (0.64, 0.73)	0.70 ± 0.07 (0.64, 0.76)	0.69 ± 0.05 (0.65, 0.73)	0.68 ± 0.05 (0.64, 0.72)
RFE LR	0.69 ± 0.04 (0.66, 0.73)	0.69 ± 0.04 (0.65, 0.73)	0.71 ± 0.05 (0.67, 0.75)	0.69 ± 0.04 (0.66, 0.73)	0.69 ± 0.04 (0.66, 0.73)
RFE RF	**0.71 ± 0.04 (0.67, 0.74)**	**0.70 ± 0.04 (0.67, 0.74)**	**0.72 ± 0.05 (0.67, 0.76)**	**0.71 ± 0.04 (0.67, 0.74)**	**0.71 ± 0.04 (0.67, 0.74)**
RFE XGB	0.686 ± 0.037 (0.65, 0.72)	0.69 ± 0.04 (0.65, 0.72)	0.69 ± 0.07 (0.64, 0.75)	0.69 ± 0.04 (0.65, 0.72)	0.69 ± 0.04 (0.65, 0.72)
Correlation	0.68 ± 0.03 (0.65, 0.71)	0.68 ± 0.04 (0.65, 0.72)	0.68 ± 0.05 (0.64, 0.72)	0.68 ± 0.03 (0.65, 0.71)	0.68 ± 0.03 (0.65, 0.71)
No selection	0.68 ± 0.03 (0.65, 0.71)	0.68 ± 0.04 (0.64, 0.72)	0.70 ± 0.08 (0.63, 0.77)	0.68 ± 0.03 (0.65, 0.71)	0.68 ± 0.04 (0.65, 0.71)

Table 4: Performance Metrics of shallow ML Models for the classification of manually-extracted features. Shown as Average ± Std (CI).

Fold	Accuracy	Precision	Recall	AUC	F1
LR	0.66 ± 0.04 (0.63, 0.69)	0.66 ± 0.04 (0.62, 0.69)	0.66 ± 0.04 (0.62, 0.70)	0.66 ± 0.04 (0.63, 0.69)	0.66 ± 0.04 (0.63, 0.69)
RF	**0.71 ± 0.03 (0.68, 0.74)**	0.70 ± 0.04 (0.67, 0.73)	**0.72 ± 0.05 (0.68, 0.77)**	**0.71 ± 0.03 (0.68, 0.74)**	**0.71 ± 0.03 (0.67, 0.74)**
XGB	0.70 ± 0.03 (0.67, 0.73)	**0.70 ± 0.04 (0.67, 0.74)**	0.71 ± 0.04 (0.67, 0.74)	0.70 ± 0.03 (0.67, 0.73)	0.70 ± 0.03 (0.67, 0.73)
Cal. XGB	0.70 ± 0.03 (0.67, 0.72)	0.70 ± 0.05 (0.66, 0.74)	0.70 ± 0.08 (0.64, 0.77)	0.69 ± 0.03 (0.667, 0.72)	0.69 ± 0.04 (0.66, 0.72)

Table 5: Performance metrics across different folds for all shallow ML models for the classification of manually-extracted features. Shown as Average ± Std (CI).

Fold	Accuracy	Precision	Recall	AUC	F1
1	**0.73 ± 0.03 (0.70, 0.76)**	**0.73 ± 0.04 (0.69, 0.77)**	**0.74 ± 0.05 (0.69, 0.78)**	**0.73 ± 0.03 (0.70, 0.76)**	**0.73 ± 0.04 (0.70, 0.76)**
2	0.68 ± 0.04 (0.65, 0.71)	0.69 ± 0.05 (0.65, 0.73)	0.65 ± 0.03 (0.62, 0.68)	0.68 ± 0.04 (0.65, 0.71)	0.68 ± 0.04 (0.65, 0.71)
3	0.67 ± 0.04 (0.64, 0.71)	0.66 ± 0.04 (0.63, 0.69)	0.72 ± 0.07 (0.66, 0.78)	0.68 ± 0.04 (0.64, 0.71)	0.67 ± 0.04 (0.64, 0.71)
4	0.69 ± 0.03 (0.67, 0.72)	0.70 ± 0.03 (0.67, 0.73)	0.67 ± 0.06 (0.63, 0.72)	0.69 ± 0.03 (0.67, 0.72)	0.69 ± 0.03 (0.67, 0.71)
5	0.67 ± 0.02 (0.65, 0.69)	0.66 ± 0.03 (0.64, 0.69)	0.71 ± 0.03 (0.69, 0.74)	0.67 ± 0.02 (0.65, 0.69)	0.67 ± 0.02 (0.65, 0.69)

Table 6: Inferred ChromAgeNet scores for aged HSC treated with compound.

Condition	Mean	Stand. Dev.	Median	Count	95% CI
Aged	0.33	0.15	0.32	260	(0.31, 0.35)
Aged + CASIN	0.40	0.18	0.37	119	(0.36, 0.43)
Aged + IOX	0.56	0.14	0.55	31	(0.51, 0.6)
Aged + RhoAi	0.48	0.19	0.47	388	(0.46, 0.5)
Aged + UNC	0.55	0.16	0.52	46	(0.5, 0.59)
Young	0.56	0.19	0.54	109	(0.52, 0.59)

Table 7: Model hyperparameters for Grid Search

Hyperparameter	LR	RF	XGBoost
C	[0.01, 0.1, 1, 10]	-	-
Penalty	['l2', 'l1']	-	-
N. Estimators	-	[100, 300, 500, 700, 900]	[100, 300, 500, 700, 900]
Max Tree Depth	-	[10, 20, 30, 40, 50]	[5, 10, 15, 20, 25]

Table 8: Mu Fidelity metrics computed across many attribution methods over 1,000 confidently and correctly predicted images for each of the training classes.

Attribution	Fidelity Young	Fidelity Aged
Saliency	0.29	0.06
DeconvNet	0.01	-0.04
GradientInput	0.03	0.01
GuidedBackprop	-0.05	0.09
IntegratedGradients	-0.17	0.00
SmoothGrad	0.03	0.00
SquareGrad	0.28	0.03
VarGrad	0.26	0.01
GradCAM	0.05	0.01
GradCAMPP	0.00	0.00
Occlusion	-0.17	0.21
Rise	0.13	0.04
SobolAttributionMethod	0.27	0.01
KernelShap	-0.21	0.06
HsicAttributionMethod	0.24	-0.06

Table 9: Distribution of confocal acquisition protocols according to the number of fluorescence channels acquired for each image

N confocal acquisition channels	Young	Aged
1 channel	188	190
2 channels	219	193
3 channels	145	168
